# Loxapine-Induced Priapism: A Case Report and Review of the Literature on Antipsychotic-Induced Priapism

**DOI:** 10.1155/2021/5589967

**Published:** 2021-07-16

**Authors:** Alexandra L. Dodd, Sunny Patel, David C. Fipps

**Affiliations:** ^1^University of South Carolina, Greenville, Prisma Health-Upstate, 701 Grove Rd, Greenville, SC 29605, USA; ^2^Mayo Clinic, 200 1st St SW Rochester, MN 55902, USA

## Abstract

In this case report, a patient with schizophrenia experienced recurrent priapism while undergoing treatment of acute psychosis. This necessitated a review of the emergent treatment of priapism and discussion of the difficulties in treating priapism in a patient with acute psychosis. Therefore, this case report explores multiple possible etiologies of priapism within the realm of psychiatric care, reviews the proposed mechanisms of medication-induced priapism, and considers the psychopharmacological concepts that pertain to antipsychotic selection in the context of antipsychotic-induced priapism.

## 1. Introduction

Penile priapism is defined as persistent erection of the penis exceeding or in the absence of sexual stimulation, usually defined as lasting greater than four hours, caused by a disruption in the physiological control of tumescence and detumescence of the penis [1]. Priapism as a side effect of psychotropic medication is of particular interest to psychiatrists, and managing this serious side effect is an important skill, especially in the acute inpatient setting. Knowledge of medication-induced priapism (MIP) is also of importance to emergency psychiatrists and consultation-liaison psychiatrists, who may be called upon to aid with psychiatric medication adjustments in a patient with priapism and comorbid psychiatric illness. This report is unique in that it describes a case of priapism associated with antipsychotic administration on an inpatient psychiatric unit and involves a medication that, to our findings, has not been previously linked with priapism. This brings forward a pertinent discussion of the pathophysiology of MIP, risk factors for priapism, and acute and long-term management of MIP, as well as an exploration of which antipsychotics may have a lower risk of MIP.

## 2. Case Description

### 2.1. Patient Information

**Figure 1:**
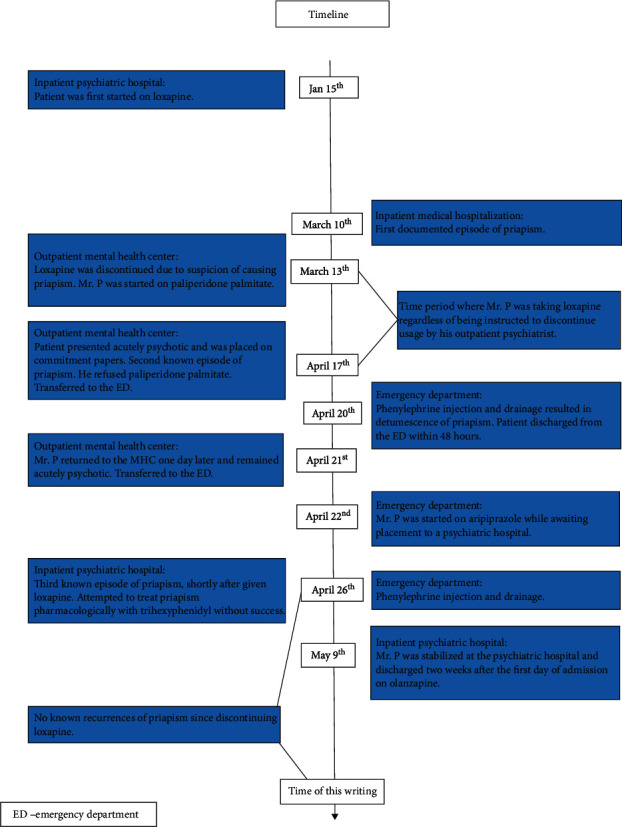
A timeline of events represented in the case report. ED = emergency department.

Mr. P is a 44-year-old single, unemployed, disabled, African American male, with a past psychiatric history of schizophrenia and cocaine use disorder, who presented to his local Mental Health Center (MHC) for a routine appointment, and was noted to be experiencing a sustained painful erection. Mr. P was noted to have a history of recurrent priapism related to past trazodone and cocaine use and no other significant medical problems. Note that any information that can be used to specifically identify the patient has been changed to ensure confidentiality. As Mr. P was seen in various medical settings, the following case report is split into three locations: Inpatient Medical Hospitalization, Mental Health Center/Emergency Department (ED), and Inpatient Psychiatric Hospital (see Figure 1 for a timeline of the clinical course).

### 2.2. Inpatient Medical Hospitalization

Mr. P presented to the ED with a chief complaint of a painful erection. He denied taking any medications for erectile dysfunction. He had been given his paliperidone palmitate long-acting injectable three days prior from the MHC. Mr. P explicitly stated that he had never experienced a sustained erection after his paliperidone palmitate injection (he had been on this medication for many months). No paliperidone level was obtained. Though trazodone 150 mg as needed for insomnia was on his home medication list, his frequency of use was unclear. Urology was consulted in the ED, diagnosed priapism, and admitted the patient. He received a phenylephrine 100 mcg/mL injection into the corpora cavernosa, resulting in successful detumescence.

During this hospitalization, the patient had a normal hemoglobin electrophoresis. Reticulocyte count indicated slight elevation at 3.5% with 0.11 million reticulocytes per microliter. Partial thromboplastin time and prothrombin time were within normal limits. Mr. P was discharged from the hospital within 24 hours.

### 2.3. Mental Health Center/Emergency Department

Four weeks later, Mr. P presented to his outpatient follow-up appointment with florid psychosis and recurrence of priapism. It was confirmed that Mr. P had discontinued his trazodone due to concerns that this medication had been the culprit of the previous episode of priapism. It appeared that he had not received any prescribed antipsychotics in four weeks, as he refused his last paliperidone injection. Mr. P was taken to the ED, where complete blood count (CBC) and comprehensive metabolic panel (CMP) were within normal limits; urine drug screen (UDS) was positive for cocaine. Urology again performed a phenylephrine injection that resulted in successful detumescence. Mr. P remained in the ED for forty-eight hours and was discharged upon resolution of acute psychosis. One day later, he presented again to the MHC exhibiting signs of acute psychosis. He was transferred to the ED, and his UDS was positive for cocaine; however, upon reaching the timeframe for full metabolism of cocaine, he remained psychotic. Four days later, after switching his antipsychotic to aripiprazole, he was transferred to an inpatient psychiatric hospital.

### 2.4. Inpatient Psychiatric Hospital

Upon arrival, Mr. P continued to experience psychosis despite treatment with aripiprazole. Records indicated during previous admissions that he responded well to loxapine 40 mg twice daily, trazodone 150 mg nightly, and lorazepam 1 mg four times daily. Mr. P was placed on the same regimen of medications, except for trazodone, given his recent episode of priapism, and aripiprazole was discontinued. One day after loxapine 40 mg twice daily per os (PO) and lorazepam 1 mg four times daily PO were started, the patient experienced another episode of priapism. The team explored possible causes of priapism, and there was strong clinical suspicion that loxapine was the culprit.

The patient was reluctant to be transferred to the ED again for another phenylephrine injection and aspiration. Given his low threshold for agitation and worsened aggression with security, noninvasive pharmacological options were considered. Upon reviewing rare case reports of M1 antagonists being used to treat MIP, trihexyphenidyl 1 mg PO was administered. This did not result in resolution of the sustained erection. After reviewing the consequences of failing to treat priapism, the patient agreed to be transferred to the ED, where he underwent another phenylephrine injection and aspiration of blood, resulting in detumescence.

Upon return to the psychiatric hospital, he was further interviewed about his pattern of loxapine use. It was discovered that his first episode of priapism occurred three months prior, shortly after discharge from the psychiatric hospitalization in which loxapine was first initiated. This raised concern at the MHC that loxapine was causing his priapism, prompting a change to paliperidone palmitate. Despite this change, Mr. P admitted to intermittently taking loxapine throughout the three-month gap between the two psychiatric hospitalizations, which appeared to correlate with his episodes of priapism. Mr. P's antipsychotic was switched to olanzapine and he continued lorazepam 1 mg four times daily. He was also started on mirtazapine 15 mg nightly for insomnia and depressive symptoms. Mr. P did not have any further episodes of priapism during this hospitalization. At the time of this writing (two years later), he has presented to the ED multiple times for psychosis, intermittently with positive sympathomimetic UDS, but no recurrence of priapism.

## 3. Discussion

Ischemic priapism is a urological emergency, as irreversible damage to tissue can occur in as little as six hours and only half of the patients treated within 24 hours regain complete premorbid function. Ischemic priapism beyond 36 hours leads predictably to lasting dysfunction [1]. Complications of priapism if not promptly treated can include damage to the corpora cavernosa, erectile dysfunction, and penile shortening secondary to fibrosis. Pharmacological treatment approaches are limited, with severe cases necessitating surgical intervention [2].

Priapism can be idiopathic or associated with a variety of etiologies, including multiple medical conditions and medications. See Table 1 for a list of potential etiological factors for priapism. Priapism is reported to result from pharmacologic agents in 20-40% of cases, and the majority of MIP cases involve either antihypertensive medication or psychotropics, specifically antipsychotics and antidepressants [3, 4]. Antipsychotic drugs are the cause of MIP in up to 50% of cases [5].

In this case, the team considered multiple potential causes of priapism. First, the team proposed that cocaine-induced priapism was unlikely considering the timeframe of cocaine metabolism had passed. Second, after a thorough review of his history, it was noted that the patient had no medical comorbidities known to contribute to priapism. Third, he was not on medications that were thought to cause priapism previously. Fourth, the timing of priapism following administration of loxapine was noted to be significant. His priapism reappeared two hours after administration of the first dosage of loxapine.

The proposed mechanism of MIP varies depending on the offending agent, but alpha-1 blockade and sympathomimetic properties are the most proposed mechanisms among all classes of drugs that can cause this unintended side effect [4]. The proposed mechanism of antipsychotic-induced priapism involves antagonism of alpha-1 adrenergic receptors by a first- or second-generation antipsychotic [11]. Higher affinity for alpha-1 adrenergic receptors has been shown to be associated with higher risk of causing priapism by inhibiting the sympathetic action that is responsible for detumescence of the penis [12]. Increasing dosage and duration of the antipsychotic does not appear to be correlated with increased risk of priapism [5]. Other factors that may increase the risk of priapism are prior history of priapism, use of multiple antipsychotics, and slow metabolism at CYP2D6, as many antipsychotics are metabolized by this enzyme.

Pharmacological and surgical treatments are aimed at resolution of the erection while preserving function of the penis. Initial conservative management of priapism can include vigorous exercise, ejaculation, applying ice packs to induce smooth muscle contraction, and pain management as needed [3, 13]. The urological consultation is a vital step in management of suspected MIP. Medical workup including CBC to assess for signs of infection or red blood cell derangement, UDS to rule out substance abuse, and in some cases a hemoglobin electrophoresis to rule out sickle cell disease are also recommended. In this case, the patient had previously undergone a negative hemoglobin electrophoresis in the workup of his priapism. A pelvic and digital rectal exam may be recommended to rule out malignancy as a potential contributing factor to the priapism [14], which was not documented in this case.

Initial surgical management involves dorsal penile nerve block with 1% lidocaine [15], and then, a large-bore needle is used to aspirate blood from the corpus cavernosum to relieve the engorgement. In the estimated one-third of cases that do not resolve with needle aspiration, next comes injection of an alpha-adrenergic agonist such as phenylephrine directly into the penis to induce vasoconstriction and penile detumescence. For rare refractory cases, further surgical intervention is needed. By this step in the intervention and for a portion of patients responding to successful earlier treatments, implantation of penile prosthesis is required to achieve future erections [3].

The invasiveness and prognosis of repeated surgical intervention for treatment of priapism necessitate earlier conservative pharmacological options, particularly in cases of MIP in which there is a theoretical target for biochemical intervention. An ideal candidate for pharmacological treatment of MIP would be fast-acting, tolerable, readily available, effective, and have action lasting longer than the offending agent, or be safe to administer in repeated doses until the offending agent has metabolized. In this case, a patient who had previously received penile phenylephrine injections for MIP initially wished to forego this procedure despite the known risks involved in failing to treat priapism.

One case report of priapism treated with benztropine resulted in resolution without surgical intervention [16]. Benztropine is a selective M1 muscarinic acetylcholine antagonist used to treat extrapyramidal side effects (EPS) [17]. Benztropine has also been used to treat non-psychotropic-associated priapism in horses, as antagonism of the parasympathetic nervous system (via cholinergic antagonism) allows detumescence [18]. Trihexyphenidyl is another similar M1 antagonist commonly prescribed for Parkinsonism and EPS [19]. Our literature review did not reveal any studies of trihexyphenidyl being used to treat antipsychotic-induced priapism. In this case, the patient had a documented allergy to benztropine, and a single dose of trihexyphenidyl unfortunately failed as pharmacological management of antipsychotic-induced priapism.

For all patients recovering from an episode of priapism, follow-up with a urologist is recommended [14]. For some patients with psychiatric illnesses, including the patient described in the case above, there may be suboptimal adherence with this recommendation. This case also brings up the question of long-term management of patients with recurrent priapism. Our literature review did not reveal any agent known to reduce the risk of recurrent antipsychotic-induced priapism. By considering the proposed mechanism of antipsychotic-induced priapism, it may be possible to target one source of the problem using existing or novel pharmacological agents and reduce the need for surgical interventions.

Antipsychotic selection may also be important in long-term management. In the case above, given that priapism is thought to be triggered by the antagonism of alpha-1 adrenergic receptors, the decision was made to treat Mr. P's psychosis with aripiprazole, which has one of the lowest affinities for alpha-1 adrenergic receptors among second-generation antipsychotic medications FDA (Food and Drug Administration) approved for schizophrenia and agitation associated with schizophrenia [20]. Aripiprazole did not control the patient's psychotic symptoms, so the antipsychotic regimen was changed to the patient's previously effective loxapine. When this change to loxapine resulted in the return of priapism, the patient's antipsychotic medication was again changed to olanzapine. Loxapine and olanzapine are structurally similar as they are both dibenzepines, though loxapine has been reported to have an affinity for the alpha-1 adrenergic receptor of 3.6 × 10^−7^ × 1/Kd, where Kd is the equilibrium dissociation constant in molarity, while olanzapine has been reported to have lower affinity for the alpha-1 receptor at 2.3 × 10^−7^ × 1/Kd. Compared to other antipsychotics such as ziprasidone, which has an affinity for the alpha-1 receptor of 38 × 10^−7^ × 1/Kd, or a tenfold difference in affinity over loxapine, the difference between olanzapine and loxapine's affinity appears marginal. Though olanzapine has been associated with priapism, the patient in this case has not had a recurrence of priapism since being prescribed olanzapine [21]. Confounding variables across numerous case reports make it difficult to directly align alpha-1 adrenergic affinity with risk of priapism, though using alpha-1 affinity may serve as a guidepost in considering selection of postpriapism therapies on an individual level.

In summary, the mainstay of treatment for priapism remains prompt implementation of the conservative management measures described above (exercise, ejaculation, applying ice packs, and pain control), accompanied by prompt consideration for surgical intervention [22]. Due to the high morbidity of priapism as a serious side effect of antipsychotic medications, clinicians should be aware of this recommended acute management of priapism and in considering the receptor profiles of medications can equip themselves to select a potentially effective medication with lower risk of recurrent priapism. There is limited data on rates of recurrence of priapism when a patient is retrialed on the offending agent, though this case report did demonstrate rapid recurrence of priapism after loxapine was restarted, in the absence of other previous risk factors attributed to the patient's presentation (acute cocaine use and recent trazodone use).

## 4. Conclusion

In this case report, loxapine was associated with recurrent MIP. The Adverse Drug Reaction Probability Scale [23] (Naranjo scale) for this case was calculated to be 6 out of 13, which indicates a probable likelihood of causality regarding loxapine inducing priapism in this patient. It was noted that knowledge of acute management of priapism, including prompt referral to emergency services and urology, is important for psychiatric physicians and that alpha-1 adrenergic affinity may be a contributing factor in antipsychotic selection after an episode of MIP, but antipsychotic efficacy will continue to be a key factor in medication selection.

## Figures and Tables

**Table 1:** Differential diagnoses, medications, and substances that have been associated with priapism.

Etiological factors for priapism	
Diagnoses	Medications/substances
(i) Amyloidosis [3]	(i) Antihypertensives [4]
(ii) Diabetes [6]	(ii) Antidepressants [4]
(iii) Gout [7]	(iii) Antipsychotics [4]
(iv) Malignancy [7]	(iv) Buprenorphine [8]
(v) Scorpion sting [3]	(v) Cocaine [3]
(vi) Sickle cell anemia [9]	(vi) Methylphenidate [4]
(vii) Spinal cord injury [7]	(vii) Synthetic cannabinoids [10]

## Data Availability

Data are available upon request.
